# Inclusive social norms and nationals’ positive intergroup orientations toward refugees: The moderating role of initial prejudice and intergroup contact

**DOI:** 10.1177/13684302231156399

**Published:** 2023-02-28

**Authors:** Giulia Valsecchi, Jacques Berent, Islam Borinca, Eva G. T. Green, Juan M. Falomir-Pichastor

**Affiliations:** 1 University of Geneva, Switzerland; 2 University College Dublin, Ireland; 3 University of Lausanne, Switzerland

**Keywords:** imagined contact, immigrants, positive intergroup orientations, refugees, social norms

## Abstract

Research on the interplay between inclusive norms and intergroup contact on improving intergroup orientations has yielded conflicting results, suggesting either that an experience of personal contact is necessary to have a positive effect of inclusive norms or that such personal experience is not always necessary. To clarify this issue, across four studies (*N* = 835), we investigated the influence of inclusive norms on positive intergroup orientations as a function of personal experiences of intergroup contact. Study 1 demonstrated that inclusive norms are more strongly correlated with experiences of personal contact with outgroups with whom opportunities of contact are more (i.e., immigrants) than less (i.e., refugees) frequent. Study 2 provided experimental evidence for this finding showing that inclusive norms increase prejudiced nationals’ willingness to engage in future contact with immigrants but not with refugees, suggesting that conformity to inclusive norms depends on varying contact opportunities with the outgroups. Studies 3 and 4 confirmed that prejudiced nationals conformed to inclusive norms specifically when experienced positive contact with a refugee (experimentally induced with the imagined contact paradigm), compared with no contact (Study 3) or negative contact (Study 4). We discuss the implications of these findings for research on intergroup contact, social influence, and intergroup relations.

In June 2021, the number of refugees and asylum seekers worldwide was estimated at 30.9 million (United Nationas High Commissioner for Refugees [UNHCR], 2021). In addition, since the start of the war in Ukraine in February 2022, over 5 million Ukrainian refugees have fled to neighboring countries (UNHCR, 2022). The growing number of refugees^1^ calls for long-term solutions to facilitate their integration into host societies and encourage harmonious relations with the host population. Despite the prevalence of egalitarian and inclusive norms in Western societies (Crandall & Eshleman, 2003; Monteith et al., 1996), contact between groups remains limited, especially between refugees and nationals (i.e., host members; Esses et al., 2017). We propose that this limited intergroup contact with refugees may decrease the influence of inclusive norms prevalent in Western societies and undermine harmonious relationships between gorups. Accordingly, in the present research, we investigate the influence of inclusive norms on nationals’ positive intergroup orientations (i.e., willingness to engage in future intergroup contact) toward refugees as a function of personal (i.e., direct or indirect) experiences of intergroup contact.

Past research has shown conflicting results, with findings suggesting that inclusive norms improve intergroup attitudes regardless of personal experiences of contact (Sechrist & Stangor, 2007; Visintin et al., 2020) or only when some degree of contact has already been personally experienced (e.g., Borinca et al., 2021; A. Kende et al., 2017; Laurence, 2014; Mähönen et al., 2011). We argue that these conflicting findings may be explained by varying opportunities for contact between the groups. In other words, nationals might have fewer opportunities to interact with refugees than with other migrant groups, such as economic immigrants, due to refugees’ lesser (or even nonexistent) integration opportunities into host societies. Therefore, fewer opportunities for contact may prevent nationals, especially those who are already resistant to intergroup interactions (i.e., prejudiced nationals), from conforming to inclusive norms promoted in Western countries. Thus, a personal experience of contact (i.e., an opportunity for contact) with refugees would be necessary to motivate prejudiced nationals to conform to inclusive norms and increase their willingness to engage in future intergroup contact.

To address this question, in one correlational and three experimental studies, we examined the role of intergroup contact in fostering prejudiced nationals’ conformity to inclusive norms. Intergroup contact was operationalized in two complementary ways: (a) by comparing two different groups with which the population has more or less personal experiences of contact, namely immigrants and refugees (Studies 1 and 2), and (b) by using an imagined contact paradigm focusing on refugees (Studies 3 and 4).

## Inclusive Norms and Intergroup Contact

Social norms are group-based standards that convey what is socially accepted and expected in a given context. The literature distinguishes two types of norms, descriptive, which refer to what most others do, and injunctive, which define what ought to be done (Cialdini et al., 1990). Conformity to descriptive norms is motivated by a desire to comply with what is considered effective or adaptive in a given situation, while conformity to injunctive norms is motivated by a desire to comply with what is morally approved by the ingroup.

In our research, we are interested in understanding the influence of inclusive norms, whether injunctive or descriptive, on positive intergroup orientations toward outgroups. For instance, national-level social norms regulate intergroup relations: Right-wing normative climates have been linked to more negative attitudes of the national population toward different outgroups (i.e., the elderly, immigrants, and women; van Assche et al., 2017), while inclusive policies are linked to less adherence to ethnic (exclusive) conceptions of the national identity (Sarrasin et al., 2020) and anti-immigrant prejudice (J. Kende et al., 2022; Visintin et al., 2018). Experimental studies also corroborate these findings by demonstrating that inclusive (vs. exclusive) norms led people to show less negative reactions toward outgroup members (Crandall & Stangor, 2008; Nesdale et al., 2005) and greater outgroup orientations (Jugert et al., 2011; Kauff et al., 2021; Tropp et al., 2014).

However, it is not clear whether the impact of inclusive norms is affected by intergroup contact opportunities. Some studies suggest that the positive effects of inclusive norms appear independently of personal contact experiences. Sechrist and Stangor (2007), for example, showed that the influence of exclusive norms on outgroup prejudice was stronger when the frequency of contact was low rather than high. However, the frequency of contact was irrelevant and outgroup attitudes were overall favorable when the norm was inclusive. Similarly, Visintin et al. (2020) demonstrated that intolerant norms increased prejudice when intergroup contact was low, but that prejudice was lower when norms were inclusive, an effect that was independent of the frequency of intergroup contact.

Other studies suggest that the positive effects of inclusive norms appear only when intergroup contact experiences are frequent and positive. In this understanding of conformity processes, inclusive norms may fail to have an effect and/or may not be perceived as appropriate or legitimate in threatening intergroup contexts (e.g., Falomir-Pichastor et al., 2004), particularly among highly identified (e.g., Falomir-Pichastor et al., 2009) and prejudiced (e.g., Falomir-Pichastor et al., 2013) ingroup members. Thus, positive contact experiences should alleviate the threat and anxiety associated with intergroup interaction (MacInnis & Page-Gould, 2015), thereby enhancing compliance with inclusive norms. Indeed, Mähönen et al. (2011) showed that Finnish secondary school boys’ conformity to inclusive family norms only emerged when they reported having positive (vs. negative) contact with immigrants. Similarly, the positive effects of inclusive institutional norms on outgroup orientations toward Roma people were stronger among Hungarian students exposed (vs. not exposed) to a Roma contact intervention (A. Kende et al., 2017). Finally, an inclusive norm improved outgroup orientations between Kosovo Albanians and Serbs only when the Kosovo Albanian majority imagined an unknown Serb offering assistance in a difficult situation (i.e., which mirrors a positive intergroup contact condition; Borinca et al., 2021).

Given the inconsistency of past findings regarding whether personal experiences of intergroup contact are a prerequisite for conformity to inclusive norms, the present research further investigates this issue by focusing on the role of the levels of contact between members of different groups as an explanatory factor. Indeed, research suggesting that the positive effects of inclusive norms are independent of personal contact experiences focuses on prejudice/attitudes and was conducted with outgroups that were overall familiar to participants (see Study 1; Sechrist & Stangor, 2007) or in diverse contexts where intergroup interactions are more frequent and more positive (Visintin et al., 2020). Conversely, research suggesting that inclusive norms positively influence outgroup orientations only when people personally experienced a positive contact with the outgroup focused on behavioral intentions and was conducted in strongly segregated and hostile intergroup contexts: Borinca et al.’s (2021) research was conducted in the postconflictual context of Kosovo where relations between Kosovo Albanians and Kosovo Serbs are still tense. Similarly, A. Kende et al.’s (2017) research was conducted in Hungary, where anti-Roma prejudice is openly expressed and the Roma community remains largely segregated. Finally, Mähönen et al.’s (2011) research was conducted in Finland, where the foreign population constitutes approximately 2.3% of the total population. Accordingly, we suggest that inclusive norms are effective at increasing positive intergroup orientations mainly toward outgroups with whom the national population have frequent (vs. less frequent) interpersonal contact.

## Direct and Imagined Intergroup Contact

Since Allport’s (1954) contact hypothesis, a large body of research has demonstrated that intergroup contact improves intergroup attitudes and reduces prejudice (for reviews, see Dovidio et al., 2017; Pettigrew & Tropp, 2006) even toward refugees. In this regard, recent research investigating the effects of a contact intervention on children’s intentions to seek a friendly contact with a refugee child showed that exposing children to a norm encouraging contact between refugees and children in the classroom increases their willingness to seek contact with a refugee child in the future (Smith & Minescu, 2022).

With respect to our main hypothesis, we suggest that inclusive norms are effective at improving intergroup orientations among adults only with groups with whom the national population has more (vs. less) frequent personal contact experiences. Consistent with this idea, MacInnis and Page-Gould (2015) investigated the paradoxical effects of intergroup interactions and intergroup contact, and proposed the idea of a contact threshold. Specifically, they argued that sporadic and low frequency interactions with unknown outgroup members may be threatening and anxiety provoking, with this anxiety leading to the avoidance of subsequent intergroup contact. However, if subsequent intergroup interactions occur, stress and anxiety are progressively reduced and, at some point (i.e., the contact threshold), they may reduce prejudice and improve intergroup orientations. This supports the idea that for outgroups with which the population has more frequent contacts—notably because the group is more present in the public sphere—inclusive norms should improve intergroup orientations. Conversely, these positive effects should be less marked for outgroups with which contact is less frequent and more sporadic.

In the present research, we tested this general hypothesis in two ways. First, we investigated the processes of conformity to social norms toward two different groups with which the national population usually has more or less personal experiences of contact, namely immigrants and refugees. Comparing these two groups allows a direct test of our hypothesis and, to our knowledge, is the first time such a comparison has been used to assess the degree of contact opportunities. Second, we focused on a low frequency contact group (i.e., refugees) and used an imagined contact paradigm as a way to experimentally test the role of personal contact experiences in the influence of inclusive norms on intergroup orientations.

### Testing the hypothesis with respect to the target group: Refugees versus immigrants

We assume that immigrants are generally more integrated into host societies than refugees are. There is evidence for this assumption in France, the country where the present research was conducted. First, the number of immigrants in France is much higher than the number of refugees: For instance, 283,237 new immigrants registered in 2020 compared to 13,927 refugees and asylum seekers (Département des statistiques, des études et de la documentation, 2022). Second, and more generally, immigrants have lived in France longer, have the right to work, and enjoy complete freedom of movement within the country, while refugees face more difficulties in integrating and their freedom of movement is limited. Indeed, refugees, upon arrival, are often placed in isolated and segregated areas away from the host population (Blank, 2019), and their freedom of movement is restricted (Mouzourakis et al., 2019). They encounter more difficulties in integrating personally and professionally, as indicated by the low percentage of refugees employed in 2019 (Barrot & Dupont, 2020; Okba, 2018). Moreover, asylum seekers—as opposed to refugees—are subject to obligations and controls within reception facilities (i.e., accommodation in reception centers and a financial allowance; Slama, 2018), do not have the right to work (i.e., they are economically dependent on state allocations; Kobelinsky, 2012), and their access to professional and basic education is very limited (Barrot & Dupont, 2020). For all these reasons, opportunities for French nationals to have personal contact experiences with asylum seekers and refugees are less frequent than those with immigrants. Whilst this situation describes the difference between immigrants and refugees in France, a similar sociopolitical situation is observed in many other EU and non-EU host countries (e.g., Alho, 2021; De Sario, 2021; Godino & Barrientos, 2021; Kapsalis et al., 2021; Liamputtong & Kurban, 2018; Shaw et al., 2021). Accordingly, in the present research, we assume that contact between nationals and refugees in host societies is less frequent compared to immigrants. Consequently, conformity to inclusive norms promoting positive intergroup relations should be greater toward immigrants than refugees.

### Testing the hypothesis with the imagined contact paradigm

Imagining intergroup contact is a technique that consists in mentally simulating a positive or negative interaction with an outgroup member. The basic assumption is that a positive simulation will create a cognitive script of a positive experience alongside with more positive attitudes toward the outgroup that will result in more favorable outgroup perceptions and positive intergroup orientations (Crisp & Turner, 2012). A number of studies have found support for this hypothesis (Crisp & Turner, 2009; Husnu & Crisp, 2010a, 2010b; Miles & Crisp, 2014; Stathi et al., 2011), and show that imagined contact improves behavioral intentions toward outgroups, such as willingness to engage in future intergroup contact (Husnu & Crisp, 2010a; Stathi et al., 2012; Turner et al., 2013; Vezzali et al., 2012) and actual behavior (Meleady & Seger, 2017).

One of the main benefits of imagined contact is its effectiveness in enhancing the anticipation of positive intergroup interactions in contexts where direct contact is less frequent and/or less desirable (Crisp & Turner, 2009), such as in segregated contexts. Previous research has notably demonstrated that imagined contact has positive effects in highly segregated contexts, such as Cyprus (Husnu & Crisp, 2010b; Ioannou, 2019), Northern Ireland, or the Arizona’s border area in the US (Paolini et al., 2014). Husnu and Crisp (2010a) showed that Turkish Cypriots who imagined having positive contact with Greek Cypriots reported greater intentions to engage in future contact with the outgroup compared to participants in a no-contact condition. Ioannou (2019), in turn, demonstrated that imagined positive contact improves Greek Cypriots’ behavioral intentions toward Turkish Cypriots.

Accordingly, the imagined contact paradigm allows overcoming the lack of personal experiences of contact with segregated outgroups: conformity to inclusive norms promoting positive intergroup relations should be observed when people have imagined a positive contact (vs. no-contact or negative contact conditions) with refugees. In other words, imagined intergroup contact should enable conformity with inclusive norms and therefore increase positive orientations toward segregated outgroups (i.e., catalyze further contact seeking; Paolini et al., 2018).

## The Moderating Role of Initial Outgroup Attitudes

Several lines of research suggest that conformity to inclusive norms is moderated by individuals’ initial attitudes toward the outgroup. Specifically, prejudiced nationals, compared to unprejudiced nationals, should benefit more from personal experiences of contact with refugees, thereby increasing their conformity to inclusive norms.

Yet, the motivation to seek outgroup contact and personal experiences of intergroup contact in the first place vary according to one’s attitude toward outgroups. Individuals with a positive (unprejudiced) attitude toward outgroups should have more contact experiences with its members, and especially with immigrants, as opposed to refugees and asylum seekers, since opportunities for contact in everyday life are less frequent with the latter groups. This assumption is also consistent with research showing that unprejudiced individuals are more open toward new experiences and are overall supportive of egalitarian values (Hodson et al., 2013; Hodson & Dhont, 2015). Conversely, prejudiced individuals are likely to avoid direct interactions with outgroup members in the first place, are less open to new experiences (Hodson et al., 2013; Hodson & Dhont, 2015), and have a greater desire to maintain social distance from outgroups (Corrigan et al., 2001). In addition, previous research has shown that positive imagined contact interventions are most effective among prejudiced individuals compared to unprejudiced individual. Because they have fewer personal experiences of contact with outgroup members they should benefit the most from simulated positive interactions (e.g., Asbrock et al., 2013; Hodson et al., 2015; West et al., 2017). Finally, previous research has shown that unprejudiced individuals are more likely to conform to inclusive norms whether or not the outgroup is perceived as threatening, but that conformity to inclusive norms is reduced among prejudiced individuals when the outgroup is perceived as threatening (Falomir-Pichastor et al., 2013).

Therefore, there is reason to believe that attitudes toward outgroups moderate the investigated processes. Overall, unprejudiced individuals have positive orientations toward outgroups, consistent with inclusive norms and their personal intergroup contact experiences. We can thus assume that unprejudiced individuals will conform to inclusive norms regardless of their personal contact experiences with a specific outgroup. Prejudiced individuals, in turn, should overall be less motivated to seek intergroup contact, and should conform to a lesser extent to inclusive norms specifically with outgroups with whom interactions are less frequent (i.e., refugees). Therefore, in the present research, we believe that priming prejudiced individuals with a positive contact experience with a refugee should promote their conformity to inclusive norms.

## Overview and Hypotheses

One correlational and three experimental studies were conducted to investigate the influence of inclusive norms on French nationals’ positive intergroup orientations toward groups with whom they have more versus less frequent contacts, and whether personal experiences of intergroup contact moderate this effect. More specifically, we expected greater conformity to inclusive norms when personal intergroup contact is high compared to low. Personal intergroup contact was operationalized in two different ways: (a) by comparing two different outgroups with whom nationals have different levels of intergroup contact (refugees and immigrants; Studies 1 and 2) and (b) by experimentally manipulating imagined intergroup contact (Studies 3 and 4).

Based on the outlined reasoning, we first wanted to empirically verify the different levels of contact that French nationals have with refugees and immigrants (Study 1). As suggested by demographic data, we expected French nationals to report less contact with refugees compared to immigrants. Moreover, based on research showing that less frequent contact in the past increases the salience of negative contact in the present (Paolini et al., 2014), we also expected to observe an effect on contact quality, with nationals reporting less positive contact experiences with refugees than with immigrants. With this prerequisite in mind, in Studies 1 and 2, we tested the prediction that inclusive (vs. exclusive) norms are more strongly related to positive intergroup orientations mainly toward outgroups with whom the host population has more frequent experiences of intergroup contact (i.e., immigrants), rather than groups with which they have less frequent experiences of contact (i.e., refugees; H1). We also predicted that imagining a positive contact with a refugee, as compared to a control condition (Study 3) or a negative contact condition (Study 4), fosters the influence of inclusive social norms on nationals’ positive intergroup orientations toward refugees (H2). Finally, the aforementioned effects should be observed specifically among prejudiced (vs. unprejudiced) nationals (H3; Studies 2, 3, and 4). Given that our general hypothesis focuses on the effects of inclusive norms in general, and that both types of norms (descriptive and injunctive) can have positive effects on positive outgroup orientations, we assessed both, inclusive injunctive (i.e., the overall perception that France promotes egalitarian values) and inclusive descriptive (i.e., the perception that French nationals have positive and frequent contact with the outgroups) norms.

## Study 1

### Method

#### Participants and procedure

Participants with French nationality were recruited on Foule Factory (an online French crowdsourcing platform) and invited to participate in an online survey about several sociopolitical issues. They were compensated with €1.60 for their time. From the initial sample of 123 participants, one participant was excluded from the analysis because s/he was binational and three because they failed the attention check, which asked them to “. . . not answer the question and simply click on the ‘Next’ button.” The final sample was composed of 119 French nationals (43.7% women; *M*_age_ = 43.56, *SD*_age_ = 13.49) of which, 74.8% were employees, 2.5% students, 5.9% did not work, 5% were unemployed, 10.1% retired, and the 1.7% remaining indicated “Other” as their professional status. A sensitivity power analysis conducted on G*Power for an ANOVA (repeated measures, within factors) revealed that our final sample was sufficient to detect a medium effect size (*f* = .09), assuming an α of .05 and a power of .80. The survey was a repeated-measures design composed of two main blocks, one focusing on immigrants and the other on refugees. Each block was presented in random order and included the same questions, repeated for each target group, while within each block all measures were presented in a fixed order. At the beginning of each block, a definition of the target group was provided. Immigrants were defined as “people who have left their country of origin voluntarily in order to carry out a professional activity or in search of better living conditions,” whereas refugees were defined as “people who have been forced to leave their country because of a threat to their physical integrity and/or because of persecution.” These definitions were also used in the subsequent studies, and were presented on the first page of the survey.

In this and all subsequent studies, demographic information was collected at the beginning of the survey, participants completed all measures in the listed order and, unless otherwise noticed, all response scales ranged from 1 (*not at all/strongly disagree*) to 7 (*absolutely/strongly agree*). At the end of the surveys, participants were thanked and debriefed. All participants provided their informed consent at the end of the study. All studies followed APA ethical guidelines and were approved by the ethics committee of the first author’s institution. Materials and data for blind peer review of all studies are available at the Open Science Framework (https://osf.io/x6mwq/?view_only=70a7d06314e941df958ad659ec455485).

#### Materials

Quantity of personal contact was assessed using Gómez et al.’s (2018) four-item scale: “How many [target group] do you have. . .” “. . . as neighbors?,” “. . . as someone you know,” “. . . as coworkers?,” “. . . as close friends?” (1 = *none*, 7 = *many*). The scores for these items were averaged to create a composite score (α = .87 and α = .79 for immigrants and refugees, respectively).

Quality of personal contact was assessed using Gómez et al.’s (2018) six-item scale: “To what extent do you consider your contact with [target group] to be. . .” “agreeable,” “balanced,” “cooperative,” “voluntary,” “as equals,” and “personally important.” *1 (not at all) to 7 (absolutely)* Items were averaged to create a composite score (α = .96 and α = .95 for immigrants and refugees, respectively).

Perceived descriptive norms were assessed through two different scales.^2^ First, we adapted the same four-item scale assessing quantity of personal contact *(1 = none, 7 = many)* by asking participants to what extent they considered that “. . . the overall French population have [target group]” “. . . as neighbors?,” “. . . as someone they know,” “. . . as coworkers?,” “. . . as close friends?”. The four items were averaged to create a composite score (α = .90 and α = .90 for immigrants and refugees, respectively). Second, we also adapted the six-item scale assessing quality of personal contact by asking participants to indicate to what extent they considered that “. . . contacts of fellow citizens with [target group] were. . .” “agreeable,” “balanced,” “cooperative,” “voluntary,” “as equals,” and “personally important.” The six items were averaged to create a composite score (α = .94 and α = .94 for immigrants and refugees, respectively).

Perceived injunctive norms were assessed by six items used in Falomir-Pichastor et al. (2013). Participants were asked to rate to what extent they thought that French nationals overall agreed with the following six statements: (a) “More efforts should be made to ensure the well-being of [target group] in society”; (b) “Do you have a positive opinion of [target group]?”; (c) “France should implement policies to ensure equality between French citizens and [target group]”; (d) “It would be fair and legitimate to condemn all forms of discrimination against [target group]”; (e) “France should adopt more favorable policies toward [target group]”; and (f) “Do you feel any concern for [target group]?” The six items were averaged to create a composite score (α = .88 and α = .90 for immigrants and refugees, respectively).

### Results

**Figure 1.**
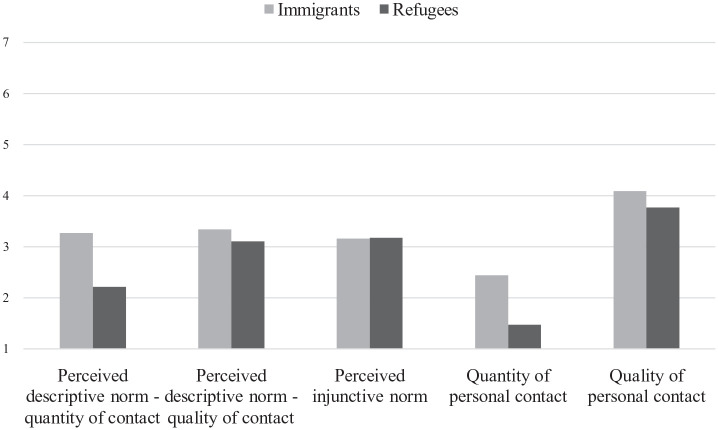
Means for perceived descriptive norms (quantity and quality), perceived injunctive norms, and personal contact (quantity and quality) with refugees and immigrants: Study 1.

Descriptive statistics and correlations between normative perceptions of intergroup contact and personal experiences of contact with the two outgroups are presented in Table 1. To empirically check whether nationals have less frequent and less positive personal contacts with refugees compared to immigrants, we conducted a repeated-measures ANOVA for each dependent variable with the target group entered as the between-subject factor (see Figure 1). To test whether perceptions of inclusive norms are more strongly related to positive personal experiences of intergroup contact with immigrants than with refugees (H1), we conducted correlational analyses for each outgroup separately, and we performed a permutation test (see Pesarin & Salmaso, 2010) to assess whether the overall correlations for refugees were less strong than the overall correlations for immigrants.

**Table 1.** Descriptive statistics and correlations for refugees/immigrants: Study 1.

	*M*	*SD*	1.	2.	3.	4.
1. Personal intergroup contact: Quantity	1.47/2.44	0.82/1.56				
2. Personal intergroup contact: Quality	3.77/4.09	1.56/1.67	.20*/.45**			
3. Perceived descriptive norms: Quantity	2.21/3.27	1.18/1.15	.50**/.62**	.25**/.47**		
4. Perceived descriptive norms: Quality	3.10/3.34	1.16/1.27	.19*/.34**	.55**/.64**	.31**/.51**	
5. Perceived injunctive norm	3.17/3.16	1.30/1.15	.01/.21*	.15/.25**	.18*/.27**	.46**/.45**

*Note. N* = 119.

* p < .05. **p < .01.

#### Personal intergroup contact

The mixed ANOVAs showed a significant main effect of target group on quantity, *F*(1, 118) = 68.49, *p* < .001, η_p_^2^ = .37, and quality, *F*(1, 118) = 9.20, *p* = .003, η_p_^2^ = .07, of personal experiences of contact. Participants reported having less frequent (*M* = 1.47, *SD* = 0.82) and less positive (*M* = 3.77, *SD* = 1.56) contacts with refugees compared to immigrants (*M* = 2.44, *SD* = 1.43; *M* = 4.09, *SD* = 1.67, for quantity and quality of contacts with immigrants, respectively).

#### Perceived descriptive norms

The ANOVAs revealed a large main effect of target group on the two scales of perceived descriptive norms: Participants perceived French fellows as having less frequent (*M* = 2.21, *SD* = 1.18) and less positive (*M* = 3.10, *SD* = 1.16) contact with refugees compared to immigrants: (*M* = 3.27, *SD* = 1.15), *F*(1, 118) = 56.97, *p* < .001, η_p_^2^ = .33 and (*M* = 3.34, *SD* = 1.27), *F*(1, 118) = 5.31, *p* = .023, η_p_^2^ = .04, for quantity and quality of contact with immigrants, respectively.

#### Perceived injunctive norms

The main effect of target group on perceived injunctive norms was not significant, indicating that, overall, participants perceived the injunctive norm as equally inclusive toward refugees (*M* = 3.17, *SD* = 1.30) and immigrants (*M* = 3.16, *SD* = 1.15), *F*(1, 118) = 0.002, *p* = .96.

### Correlational Analyses

All correlations between personal experiences and normative perceptions of intergroup contact were higher for immigrants than for refugees. Furthermore, all correlations were significant for both groups except for the correlations between perceived injunctive norms and the quantity and quality of personal contact for refugees (*r* = .07, *p* > .37 and *r* = .15, *p* = .11, for quantity and quality of contact, respectively), while these correlations were significant for immigrants (*r* = .17, *p* = .039 and *r* = .25, *p* < .01, for quantity and quality of contact, respectively).

No ready-made procedure allows to test a fully within-subjects design; therefore, we performed a permutation test^3^ to investigate whether correlations between normative perceptions and intergroup contact differed significantly between the two groups. A permutation test (also called rerandomization test) makes use of the proof by contradiction and tests the null hypothesis that all samples come from the same distribution (Onghena, 2018). For our research, we summed the test statistics for the correlations (*z*-values for x, y, and z) and assessed their significance under the null hypothesis predicting that there is no difference in the correlations between refugees and immigrants (see Pesarin & Salmaso, 2010). To generate the null distribution of this statistic, we constructed permuted samples (i.e., a rerandomized sample) where, for each sample, the refugee and immigrant scores of a given participant were either reversed (refugee scores are replaced by immigrant scores, and immigrant scores are replaced by refugee scores) or unchanged. The same statistic was calculated on each of the permuted samples. This analysis indicated that, overall, correlations between normative perceptions and personal contact experiences were less strong for refugees than for immigrants, *z* = −9.35, *p* = .003.

### Discussion

Consistent with demographic data, we found that French nationals have fewer and less positive experiences of contacts with refugees than with immigrants. Exploratory analyses on descriptive norms replicated these findings, indicating that nationals perceive their French fellows as having less frequent and less positive experiences of contacts with refugees than with immigrants. Finally, and in accordance with findings suggesting that Western societies promote egalitarian norms (Crandall & Eshleman, 2003), participants perceived injunctive norms to be equally inclusive toward refugees and immigrants. Overall, Study 1 suggests that personal experiences of intergroup contact are less frequent and less positive with refugees compared to immigrants, despite participants perceiving similar injunctive norms for both groups.

More importantly, comparisons between correlations provide preliminary evidence in support of H1. Indeed, the link between normative perceptions and personal contact experiences is stronger for immigrants than for refugees. Interestingly, the correlations between norms and personal contact with refugees were weaker overall but still significant, with the exception of the two correlations with injunctive norms. Thus, contact with refugees appears to be unrelated to injunctive norms, despite these are perceived to be inclusive. Considering that injunctive norms are generally more strongly related to prejudice reduction (Crandall et al., 2013) and interpersonally oriented forms of self-awareness (Jacobson et al., 2011), compared to descriptive norms, these findigns are highly relevant and adressed in Study 2.

The correlational nature of this study does not permit causal conclusions, which limits the confidence in our interpretation of conformity processes. Study 2 was performed to overcome this limitation.

## Study 2

The main objective of Study 2 was to experimentally test H1 and investigate the moderating role of initial outgroup attitudes. According to our rationale, we expect inclusive (vs. exclusive) norms to increase positive intergroup orientations specifically toward outgroups with whom nationals have more (vs. less) frequent experiences of contact (i.e., with immigrants more than with refugees; H1). These effects should be specifically observed among prejudiced nationals (H3). In Study 2, we focused solely on injunctive norms, since injunctive norms in particular do not seem to influence contact experiences with refugees, whereas they do for immigrants (see Study 1).

We employed a quasi-experimental design where we assessed participants’ initial attitudes toward the outgroup as an individual difference, and then experimentally manipulated the prevailing injunctive norm (exclusive vs. inclusive) and the target outgroup (refugees vs. immigrants).

### Method

#### Participants and procedure

As in Study 1, participants of French nationality were recruited on Foule Factory. They were compensated with €2.87 for their time. From the initial sample of 314 participants, 61 were excluded from the analysis because they indicated having previously participated in a similar survey, two because they were binational, and seven because they failed the attention check. The final sample was composed of 244 French nationals (51.6% women; *M*_age_ = 36.96, *SD*_age_ = 11.49) of which, 65.6% were employees, 10.2% students, 10.2% did not work, 7.8% were unemployed, and the 6.1% remaining were retired. A sensitivity power analysis conducted on G*Power for an ANCOVA (fixed effects, main effects, and interactions) revealed that our final sample was sufficient to detect a small effect size (*f* = .17), assuming an α of .05 and a power of .80. After being randomly assigned to one of the two target group conditions (immigrants vs. refugees), participants rated their attitudes toward the target group, were randomly assigned to one of the two experimental norm conditions (inclusive vs. exclusive) and finally rated their intergroup orientations toward the outgroup.

### Materials

#### Independent variables

##### Target group

Participants were randomly assigned to one of the two target group conditions. Those in the refugees target group condition completed a survey in which all information referred to refugees, while those in the immigrants target group condition completed the same survey but referring to immigrants. Thus, with the exception of the target group, the surveys were identical.

Initial attitude toward target group was assessed using a three-item scale: “Living conditions of [target group] currently living in France should be improved,” “The French government should implement measures to ensure equal rights between French and [target group],” and “The French government should adopt a more favorable policies toward [target group].” The three items were averaged into a single initial attitude scale (*M* = 2.14, *SD* = 2.33; α = .89). Higher scores represent more positive attitudes toward the target group.

The injunctive norm was manipulated by adapting the six items used in Study 1 to the target group. Participants were given the results of a bogus survey allegedly conducted on a representative sample of French nationals. The results were displayed in terms of percentage of responses (i.e., “Yes,” “I don’t know,” and “No”) to the following questions: (a) “More effort should be made to ensure the well-being of [target group] in society”; (b) “Do you have a positive opinion of [target group]?”; (c) “France should implementput policies to ensure equality between French citizens and [target group]”; (d) “It would be fair and legitimate to condemn all forms of discrimination against [target group]”; (e) “France should adopt more favorable policies toward [target group]”; and (f) “Do you feel any concern for [target group]?” In the exclusive norm condition, higher percentages were associated with exclusive responses (i.e., 88, 78, 75, 80, 90, and 70% for the “No” answer, respectively); whereas the reverse was true for the inclusive norm condition.

#### Dependent variables

##### Manipulation check

Right after the ingroup norm manipulation, participants had to indicate whether, according to the results of the survey, the attitude of the French population toward the target group was negative (1) versus positive (7) (*M* = 3.50, *SD* = 2.35).

Positive intergroup orientations were assessed using a three-item scale referring to participants’ desire to interact with outgroup members in the future: “Would you be in favor of interacting with [target group] in general?”; “Would you be willing to attend places also attended by [target group]?”; and “Could you imagine spending time with [target group] in the future?” Items were averaged to create a single Willingness for Intergroup Contact Scale (*M* = 4.93, *SD* = 1.74; α = .99).

### Results

To test our hypotheses, all dependent variables were submitted to a full-factorial ANCOVA in which the target group (immigrants = −1, refugees = +1) and injunctive norm (exclusive = −1, inclusive = +1) were introduced as independent variables; initial attitudes toward the target group (standardized scores) were considered as a moderator and introduced as a supplementary independent variable. For all analyses, we defined a model testing all the main effects as well as all (first- and second-order) interaction effects.

#### Manipulation check

A large main effect of the norm emerged on the check measure, *F*(1, 236) = 300.57, *p* < .001, η_p_^2^ = .56. As expected, participants in the inclusive norm condition perceived the majority of their ingroup as having more positive attitudes toward the outgroup (*M* = 5.37, *SD* = 1.79) than participants in the exclusive norm condition (*M* = 1.84, *SD* = 1.30). No other effect reached significance, all *F*s(1, 236) < 3.21, all *p*s > .07.

#### Positive intergroup orientations

A significant main effect of participants’ initial attitude emerged such that positive intergroup orientations increased as positive initial attitudes toward the target group increased (β = .17), *F*(1, 236) = 154.00, *p* < .001, η_p_^2^ = .40. The main effect of target group was also significant, *F*(1, 236) = 21.40, *p* < .001, η_p_^2^ = .08; participants had less positive intergroup orientations toward refugees (*M* = 4.63, *SD* = 1.77) than toward immigrants (*M* = 5.23, *SD* = 1.66). The main effect of the norm was not significant, *F*(1, 236) = 0.55, *p* = .46.

**Figure 2.**
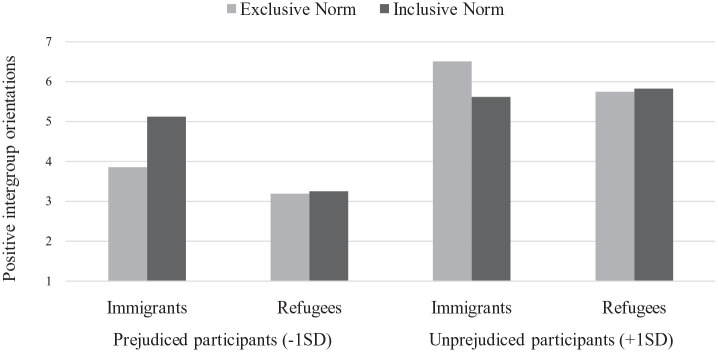
Estimated means for positive intergroup orientations as a function of target group (immigrants, refugees), ingroup norms (exclusive, inclusive), and participants’ initial attitude toward the target group: Study 2.

The two-way interactions between initial attitude and the norm, *F*(1, 236) = 10.37, *p* < .001, η_p_^2^ = .04, and between initial attitude and target group, *F*(1, 236) = 8.83, *p* = .003, η_p_^2^ = .04, were significant. These effects were qualified by the predicted Initial Attitude × Injunctive Norm × Target Group interaction, *F*(1, 236) = 10.79, *p* = .001, η_p_^2^ = .04 (see Figure 2). The interaction between the norm and the target group was significant among unprejudiced nationals (+1 *SD* above the mean on the Initial Attitude Scale), *F*(1, 236) = 4.31, *p* = .039, η_p_^2^ = .03. Specifically, exclusive (vs. inclusive) norms increased unprejudiced nationals’ positive intergroup orientations toward immigrants, *F*(1, 236) = 6.73, *p* = .01, η_p_^2^ = .03, but had no effect on orientations toward refugees, *F*(1, 236) = 0.06, *p* = .82. More importantly, the interaction between the norm and the target group was also significant among prejudiced nationals (−1 *SD* below the mean on the Initial Attitude Scale), *F*(1, 236) = 6.65, *p* = .011, η_p_^2^ = .03. Specifically, inclusive (vs. exclusive) norms increased prejudiced nationals’ positive intergroup orientations toward immigrants, *F*(1, 236) = 16.53, *p* < .001, η_p_^2^ = .06, but had no effect on orientations toward refugees, *F*(1, 236) = 0.02, *p* = .88.

### Discussion

Results of Study 2 showed that participants had more positive intergroup orientations toward immigrants than toward refugees, which is consistent with recent social surveys (Holme & Prudhomme, 2016). Surprisingly, results also showed that exclusive (vs. inclusive) norms increased unprejudiced nationals’ positive intergroup orientations toward immigrants. This unexpected finding is nevertheless consistent with research showing that counter-conformity to social norms can occur when they challenge individuals’ personal values (Falomir-Pichastor et al., 2013).

More importantly, and consistent with H1 and H3, inclusive (vs. exclusive) norms increased prejudiced nationals’ positive intergroup orientations toward immigrants, but not toward refugees. Indeed, we expected prejudiced nationals to conform less to inclusive norms in the refugee outgroup condition, namely because personal experiences of contact with refugees are overall less frequent than with immigrants. Thus, Study 2 allowed us to confirm and extend past findings by showing (a) that when personal experiences of contact are low, injunctive norms fail to improve intergroup relations and (b) that these effects are moderated by participants’ initial attitudes toward the outgroup. One may argue that the effects of the norm are driven by the exclusive (rather than the inclusive) experimental condition. Thus, a baseline/control norm condition would allow us to further confirm the specific effects of inclusive (vs. exclusive) injunctive norms.

Moreover, and given that H1 was based on a comparison between two outgroups, one may still argue that the observed effects may not be exclusively driven by nationals’ personal experiences of intergroup contact, but by the mere (de)alignment between descriptive and injunctive norms. Indeed, overall, nationals perceive French fellows as having fewer and less positive contact with refugees compared to immigrants (descriptive norm) but perceive injunctive norms as equally inclusive toward both groups. Thus, descriptive and injunctive norms are more aligned for immigrants than refugees. In this regard, recent research demonstrated that conformity to injunctive norms (i.e., an inclusive norm encouraging intergroup contact) is greater when descriptive norms are aligned (i.e., when perceived intergroup contact is high; Gómez et al., 2018; see also Cialdini et al., 1991; Reno et al., 1993). To rule out this alternative, and to test more directly our hypothesis as to whether personal experiences of contact with outgroup members—rather than perceptions of descriptive norms—explain conformity to inclusive norms, in the next studies, we solely focused on refugees and experimentally manipulated nationals’ experience of contact with refugees via the imagined contact paradigm.

## Study 3

In Study 3, we investigated whether imagining a positive contact with a refugee (vs. a control contact condition) enhances the influence of inclusive norms on nationals’ positive intergroup orientations toward refugees (H2). Again, this effect should be observed specifically among prejudiced nationals (H3). Moreover, we included a baseline/control norm condition to confirm the specific effects of inclusive norms on increasing positive intergroup orientations. Thus, we tested a linear hypothesis according to which the control norm condition lies between the inclusive and exclusive experimental conditions. We employed a quasi-experimental design where we assessed participants’ initial attitude toward refugees at the beginning of the survey, and then experimentally manipulated both injunctive norms (exclusive vs. control vs. inclusive) and imagined contact (positive vs. control).

### Method

#### Participants and procedure

We again recruited participants with French nationality on Foule Factory. They were compensated with €2.87 for their time. From an initial sample of 290 participants, nine were removed from the analysis because they were binational and two because they failed the attention check (see Studies 1 and 2). In addition, and to ensure that participants who took part in Study 2 did not participate in this study, we excluded 36 participants that indicated having previously participated in a similar survey. The final sample was composed of 243 French nationals (51.6% women; *M*_age_ = 38.06, *SD*_age_ = 11.43) of which, 65% were employees, 10.7% students, 2.5% students and employees, 7.4% did not work, 6.6% were unemployed, 4.1% were retired, and 3.7% indicated “Other.” A sensitivity power analysis conducted on G*Power for an ANCOVA (fixed effects, main effects, and interactions) revealed that our final sample was sufficient to detect a small to medium effect size (*f* = .80), assuming an α of .05 and a power of .80. Participants had firstly to rate their attitudes toward refugees and were randomly assigned to one of the six experimental conditions in a 2 (imagined contact: positive, control) × 3 (injunctive norm: inclusive, control, exclusive) between-subjects design.

#### Independent variables

Initial attitude toward refugees was assessed with a single item: “What is your attitude toward refugees?” (1 = *very negative*, 7 = *very positive*). Higher scores indicate a more positive attitude (*M* = 4.99, *SD* = 1.46).

The injunctive norm manipulation was operationalized as in the refugee condition of Study 2. Moreover, we introduced a control condition without normative information, in which participants were automatically redirected to the rest of the study.

Imagined contact was manipulated following previous research (Crisp & Turner, 2009; Husnu & Crisp, 2010b). Participants were asked to imagine a positive interaction with a refugee as follows:

We would like you to imagine that you are sitting in the waiting room of your doctor’s office and the person sitting next to you starts a conversation. You don’t know this person, but as the conversation progresses, you learn that this person is a refugee. The friendly tone of voice, open posture, and friendly expression put you at ease. The conversation is pleasant and relaxed and goes on for 20 minutes in a very warm atmosphere.

After the imagination task, participants were asked to write in a few words what they imagined. No information was presented in the control/baseline condition.

#### Dependent variables

##### Manipulation check

As in Study 2, participants were asked to rate the attitude of the French population toward refugees, as reported in the norm manipulation (1 = *negative*, 7 = *positive; M* = 3.92, *SD* = 2.46). Because this question assessed comprehension of the norm manipulation, only participants in the inclusive and exclusive norm conditions answered this manipulation check.

Positive intergroup orientations were assessed as in Study 2 but solely focused on refugees (*M* = 5.18, *SD* = 1.62; α = .95).

### Results

#### Manipulation check

Since participants in the control norm condition did not answer this set of questions, they were excluded from this analysis. Thus, we performed a full-factorial ANCOVA where injunctive norms (inclusive = +1, exclusive = −1) and imagined contact (positive = +1, control = −1) were introduced as independent variables. Participants’ initial attitude toward refugees (standardized scores) was considered as a moderator and introduced as a supplementary independent variable. We tested a model including all the main effects as well as the first- and second-order interaction effects. The analysis revealed a significant main effect of the norm manipulation, *F*(1, 149) = 209.14, *p* < .001, η_p_^2^ = .60. As expected, participants in the inclusive norm condition indicated that the majority of the French population had a more positive attitude toward refugees (*M* = 5.87, *SD* = 1.39) compared to participants in the exclusive norm condition (*M* = 1.94, *SD* = 1.57). No other effect reached significance, all *F*s < 2.21, all *p*s > .14.

#### Positive intergroup orientations

The three levels of the manipulated injunctive norm variable were broken down into two contrasts, since contrast analyses are more precise for assessing the effects of a variable with more than two modalities (Brauer & McClelland, 2005; Furr & Rosenthal, 2003). According to our hypotheses, the critical contrast tested the linear effect between the three experimental conditions by opposing the inclusive norm condition to the exclusive norm condition, with the baseline/control condition situated in between (C1: exclusive = −1, control = 0, inclusive = +1). The orthogonal contrast tested the residual variance by opposing the two norm conditions to the control condition (C2: exclusive and inclusive = −1, control = 2). If our hypothesis is confirmed, we expect C1 to be significant and C2 to be not significant.^4^ To test H2 and H3, the two orthogonal contrasts, imagined contact (control = −1, positive contact = +1), and participants’ initial attitudes toward refugees (standardized values) as well as the interactions between these terms (except for the interaction between contrasts) were entered as independent variables in a full-factorial ANCOVA.

**Figure 3.**
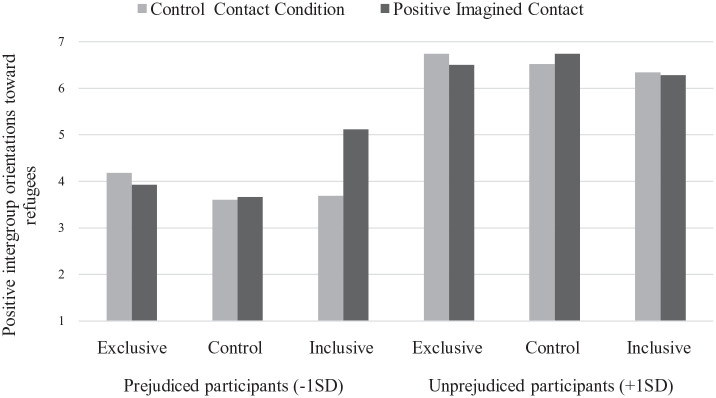
Estimated means for positive intergroup orientations toward refugees as a function of injunctive norms (exclusive, control, inclusive), imagined contact (control, positive), and participants’ initial attitude toward refugees: Study 3.

The analysis revealed a significant main effect of initial attitude toward refugees; participants’ positive intergroup orientations increased as initial attitude was more positive (β = 1.25), *F*(1, 231) = 351.41, *p* < .001, η_p_^2^ = .60. The Imagined Contact × C1 interaction was also significant, *F*(1, 231) = 7.85, *p* = .006, η_p_^2^ = .03. This two-way interaction was qualified by the predicted Initial Attitude × Imagined Contact × C1 interaction, *F*(1, 231) = 4.82, *p* = .029, η_p_^2^ = .02, while the Initial Attitude × Imagined Contact × C2 interaction was not significant, *F*(1, 231) = 2.68, *p* = .103, η_p_^2^ = .01 (see Figure 3). Simple effects revealed that the C1 × Imagined Contact interaction was only significant among prejudiced nationals (−1 *SD*), *F*(1, 231) = 10.86, *p* < .001, η_p_^2^ = .05. As expected, in the inclusive norm condition, prejudiced nationals’ intergroup orientations were more positive in the positive, compared to the control, imagined contact condition, *F*(1, 231) = 11.63, *p* < .01, η_p_^2^ = .05. The effect of the imagined contact manipulation was not significant in the exclusive norm condition, *F*(1, 231) = 0.75, *p* = .39. No other effect reached significance, all *F*s < 2.78, all *p*s > .10.

### Discussion

Results of Study 3 provided evidence in support of H2 and H3. Prejudiced nationals conformed to inclusive (vs. exclusive) norms by increasing positive intergroup orientations toward refugees solely after imagining a positive interaction with a refugee. This effect was not observed in the control condition (without imagined contact) nor among unprejudiced nationals.

Some methodological limitations need to be mentioned. First, the control no-contact condition deviates from the usual control conditions in imagined contact research where respondents are invited to complete an imagination task unrelated to the focus of the study. Indeed, the usual control conditions may include nonrelevant positive interactions, outgroup priming, neutral contact, and no-contact control scenes (e.g., Stathi et al., 2012; Turner et al., 2007), and are crucial to rule out positive affect arising from generalized social interaction as an explanation for imagined contact effects (Crisp & Turner, 2012). A second important limitation of this study is the length of survey completion for those exposed to the no-contact, no-norm conditions relative to the other conditions. Indeed, those who were exposed to the control conditions most likely took a much shorter time to complete the survey, which may have resulted in better response quality (Gibson & Bowling, 2020).

## Study 4

Study 4 was designed to replicate the findings of Study 3 while overcoming some of the methodological limitations. First, we reintroduced the three-item scale used in Study 2 to assess participants’ initial attitudes toward refugees. Second, while in Study 3 we compared a positive imagined contact condition to a control (no-contact) condition, which could have biased the responses due to the length of the study and the unusual nature of the no-contact control condition, we now explored the effects of a negative imagined contact compared to a positive imagined contact. Past research has shown that negative contact can have negative (Barlow et al., 2012), positive (Harwood et al., 2017), or no effects on intergroup relations (Joyce & Harwood, 2014). Furthermore, Dhont and van Hiel (2009) demonstrated that the negative effects of intergroup contact are particularly strong among prejudiced individuals. These mixed results encouraged us to further investigate the effects of negative contact, especially given that little research has investigated these effects in an imagined contact setting. Finally, because results of Study 3 showed that the control (without norm) and exclusive norm conditions produced the same effects (see Endnote 4), we only compared an inclusive norm condition to an exclusive norm condition. We expect that positive (vs. negative) imagined contact with a refugee will enhance the influence of inclusive norms on nationals’ positive intergroup orientations toward refugees (H2). Again, this effect should be observed specifically among prejudiced nationals (H3).

### Method

#### Participants and procedure

We recruited participants with French nationality on Foule Factory. They were compensated with €2.22 for their time. From the initial sample of 308 participants, 10 were removed from the analysis because they were binational and four because they did not provide a correct answer to the attention check (see previous studies). In addition, and to ensure that participants who took part in Studies 2 and 3 did not participate in this study, we excluded 65 participants that reported having participated in a similar survey. The final sample was composed of 229 French nationals (57.4% women; *M*_age_ = 36.95, *SD*_age_ = 11.72) of which, 65.1% were employees, 9.6% students, 1.3% students and employees, 10.0% did not work, 8.8% were unemployed, 4.8% were retired, and one person did not report his professional status. A sensitivity power analysis conducted on G*Power for an ANCOVA (fixed effects, main effects, and interactions) revealed that our final sample was sufficient to detect a small effect size (*f* = .80), assuming an α of .05 and a power of .80. Participants first rated their attitudes toward refugees and were randomly assigned to one of the four experimental conditions in a 2 (contact: positive, negative) × 2 (injunctive norm: exclusive, inclusive) between-subjects design.

#### Independent variables

Initial attitudes toward refugees were assessed with the three-item scale used in Study 2 (*M* = 4.29, *SD* = 1.67; α = .88). The injunctive norm was also induced as in Study 3, except that only the inclusive and exclusive norm conditions were presented.

##### Imagined contact manipulation

Positive imagined contact was manipulated as in Study 3. In the negative contact condition, participants were asked to:

Imagine that you are sitting in the waiting room of your doctor’s office and the person sitting next to you starts a conversation. You don’t know this person, but as the conversation progresses, you learn that this person is a refugee. The unpleasant tone of voice, closed posture, and unfriendly expression make you uncomfortable. The conversation, unpleasant and tense, takes place for 20 minutes in a very cold atmosphere.

After the imagination task, participants were asked to write in a few words what they imagined.

#### Dependent variables

##### Manipulation checks

The ingroup norm check was the same as in the previous studies (*M* = 4.24, *SD* = 2.54). Following the imagined contact manipulation, participants were asked to rate on a 7-point scale, “How would you describe the interaction you imagined with the person in the waiting room?” (1 = *very negative*, 7 = *very positive*) and “How did you find the person you imagined during this interaction?” (1 = *very unpleasant*, 7 = *very pleasant*). These two items were averaged to create a single Imagined Contact Check Scale (*M* = 4.46, *SD* = 1.85; *r* = .93).

Positive intergroup orientations were assessed as in Studies 2 and 3 (*M* = 4.86, *SD* = 1.75; α = .96).

### Results and Discussion

To test our main hypothesis, all dependent variables were submitted to a full-factorial ANCOVA in which ingroup norm (exclusive = −1, inclusive = +1) and imagined contact (negative = −1, positive = +1) were introduced as independent variables. Participants’ initial attitude toward refugees (standardized scores) was considered as a moderator and introduced as a supplementary independent variable. For all analyses, we tested a model including all the main effects as well as all (first- and second-order) interaction effects.

#### Manipulation checks

The full-factorial ANCOVA on the norm check revealed a large main effect of the norm manipulation, *F*(1, 221) = 701.53, *p* < .001, η_p_^2^ = .76. Overall, participants in the inclusive norm condition perceived the norm as more inclusive (*M* = 6.38, *SD* = 0.15) than participants in the exclusive norm condition (*M* = 1.80, *SD* = 0.16). Moreover, the Initial Attitude × Injunctive Norm interaction was also significant, *F*(1, 221) = 6.62, *p* = .011, η_p_^2^ = .03. Prejudiced nationals rated the norm as being more positive in the inclusive norm condition (*M* = 6.12) compared to the exclusive norm condition (*M* = 2.09), *F*(1, 221) = 43.44,*p* < .001, η_p_^2^ = .16. This effect was stronger among unprejudiced participants (*M* = 6.38 and *M* = 1.49, for the inclusive and exclusive ingroup norm, respectively); *F*(1, 221) = 75.54, *p* < .001, η_p_^2^ = .26. No other effects reached significance, all *F*s < 1, all *p*s > .80.

Analyses on the imagined contact manipulation check revealed a significant main effect of participants’ initial attitude, *F*(1, 221) = 75.82, *p* < .001, η_p_^2^ = .25. Overall, imagined contact was perceived as more positive as participants’ positive attitude increased (β = .47). The main effect of imagined contact was also significant, *F*(1, 221) = 672.27, *p* < .001, η_p_^2^ = .75. As expected, participants in the positive contact condition imagined a more positive interaction (*M* = 5.82, *SD* = 0.99) than participants in the negative contact condition (*M* = 2.76, *SD* = 1.11). Finally, the analysis also revealed a main effect of the ingroup norm, *F*(1, 221) = 5.79, *p* = .017, η_p_^2^ = .03. Participants in the inclusive norm condition perceived the interaction as being more positive (*M* = 6.02, *SD* = 1.87) than participants in the exclusive norm condition (*M* = 5.72, *SD* = 1.81). No other effects reached significance, all *F*s(1, 221) < 1, all *p*s > .40.

#### Positive intergroup orientations

**Figure 4.**
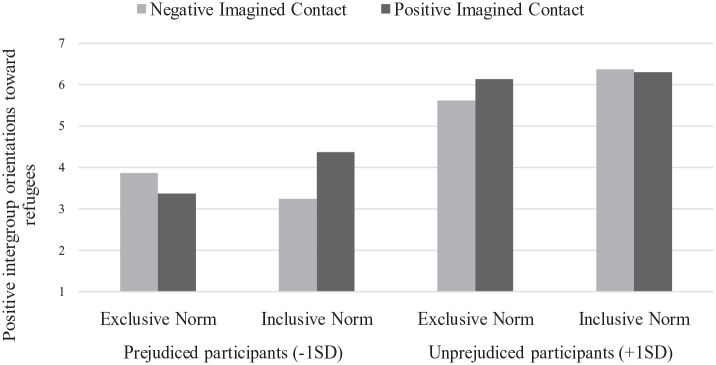
Estimated means for positive intergroup orientations toward refugees as a function of injunctive norms (exclusive, inclusive), imagined contact (negative, positive), and participants’ initial attitudes toward refugees: Study 4.

The full-factorial ANCOVA on the main dependent variable showed a significant main effect of participants’ initial attitude: Positive intergroup orientations increased as initial positive attitudes toward refugees increased (β = .95), *F*(1, 221) = 169.68, *p* < .001, η_p_^2^ = .43. The analysis also revealed the predicted Initial Attitude × Injunctive Norm × Imagined Contact interaction, *F*(1, 221) = 7.64, *p* = .006, η_p_^2^ = .03 (see Figure 4). Simple effects showed that the Injunctive Norm × Imagined Contact interaction was not significant among unprejudiced nationals (+1 *SD*), *F*(1, 221) = 1.23, *p* = .27, but it was among prejudiced nationals (−1 *SD*), *F*(1, 221) = 7.12, *p* = .008, η_p_^2^ = .03. As expected, in the inclusive norm condition, prejudiced nationals’ intergroup orientations were more positive in the positive, compared to the negative, imagined contact condition, *F*(1, 221) = 10.14, *p* = .002, η_p_^2^ = .04. The imagined contact manipulation had no effect in the exclusive norm condition, *F*(1, 221) = 0.67, *p* = .41. No other main or interaction effects were significant, all *F*s(1, 221) < 3.5, all *p*s > .07.

Results of Study 4 confirmed those observed in Study 3 and provided consistent evidence in support of H2 and H3. Specifically, positive (vs. negative) imagined contact increased prejudiced nationals’ positive intergroup orientations toward refugees when the norm was inclusive, suggesting that positive imagined contact fosters the influence of inclusive injunctive norms. As for the effects of negative contact, it remains difficult to determine whether it has negative effects or no effect on intergroup orientations, as only a control condition could have located the baseline. However, we can affirm that, in our studies, the negative contact condition did not produce positive effects on intergroup relations (see Harwood et al., 2017).

## General Discussion

Past research has shown inconsistent findings regarding the effects of intergroup contact on conformity to injunctive norms. Indeed, some studies suggest that inclusive norms improve intergroup attitudes regardless of personal or intergroup contact experiences (Sechrist & Stangor, 2007; Visintin et al., 2020), while others suggest that inclusive norms improve intergroup attitudes only when people have already experienced some form of positive intergroup contact (e.g., Borinca et al., 2021; A. Kende et al., 2017; Mähönen et al., 2011). To gain insight into when inclusive norms improve intergroup relations with regard to personal experiences of intergroup contact, in one correlational and three experimental studies, we examined the role of intergroup contact in fostering conformity to inclusive norms. We specifically focused on prejudiced nationals, since prejudiced people are likelier to avoid direct interactions in the first place, are less open to new experiences (Hodson et al., 2013; Hodson & Dhont, 2015), and have a greater desire to maintain social distance from outgroups (Corrigan et al., 2001). In other words, prejudiced people are those most in need of intervention to improve intergroup relations. Conversely, unprejudiced nationals should overall have positive outgroup orientations irrespective of norms and contact.

The intergroup contact intervention was operationalized in two complementary ways: (a) by comparing two different groups with which the population has more or less personal experiences of contact, namely immigrants and refugees, and (b) by using the imagined contact paradigm focusing on refugees. All studies were conducted in France, where refugees are overall less integrated compared to other migrant groups.

Study 1 aligned with demographic data, showing that nationals’ contact with refugees is less frequent and less positive as compared to contact with immigrants. This finding converged with the perceived descriptive norm: individuals perceive French fellows as having less frequent and less positive contacts with refugees as compared to those with immigrants. However, the perceived injunctive norm was similar for both groups: individuals perceived both groups as equally protected by an inclusive norm. Given the difference in past contact experiences, we expected conformity to inclusive norms promoting positive intergroup relations to be greater toward immigrants than refugees (H1). Providing preliminary correlational evidence to support this hypothesis, Study 1 showed that, overall, normative perceptions were more strongly related to personal contact experiences with immigrants than with refugees. Furthermore, the only situation in which normative perceptions were not related to personal intergroup contact experiences with refugees was in the case of injunctive norms, which led us to specifically manipulate this type of norm in subsequent studies.

Study 2 experimentally confirmed these results and showed that inclusive (vs. exclusive) norms increased prejudiced nationals’ positive intergroup orientations toward immigrants, but not toward refugees (H1 and H3). In other words, inclusive norms appeared to foster intergroup relations among groups with whom the national population has higher personal experiences of contact (immigrants), but not for groups with whom they have lower personal experiences of contact (refugees). In Studies 3 and 4, we focused solely on refugees, as we wanted to experimentally test the unique role of personal contact experiences in shaping conformity to injunctive norms. Intergroup contact was operationalized with the imagined contact paradigm. Again, we demonstrated that conformity to inclusive norms among prejudiced nationals only emerges when participants have imagined a positive, as compared to a control (Study 3) or negative (Study 4), contact with a refugee (H2 and H3).

The present research makes a novel contribution to the understanding of the contexts in which inclusive norms improve intergroup orientations and shows that experiences of personal contact (whether real or imagined) are required to increase conformity to inclusive norms. These findings also highlight the relevance of imagined contact as a strategy to encourage conformity to inclusive norms when direct personal contact is low.

### Limitations and Future Research Directions

Despite the contributions of the studies, some limitations call for discussion. As noted earlier, personal contact experiences were operationalized in two complementary ways: by varying the target outgroup (immigrants vs. refugees) and by experimentally manipulating the imagined contact. While these two different methodological approaches increase the relevance and strength of the observed results by providing consistent evidence, they may also constitute a limitation in that they refer to two different levels of analysis. Indeed, varying the target group refers to a macro-level analysis where the overall frequency of contact between the national population and the two target groups is at stake. Conversely, the imagined contact paradigm refers to a micro-level analysis where the (imagined) interaction specifically took place between the participant and the outgroup member. In the micro-level approach, the self was directly involved in the interaction and the participant was asked to actively simulate a contact experience (White et al., 2021). The macro level, in turn, did not consider the intergroup contact of each participant, which could have oriented them to consider intergroup relations, stereotypes, and norms more generally, beyond their personal experiences. Despite this limitation, it is worth noting that Study 1 combined both levels of analysis by showing that participants in our research had fewer personal experiences of contact with refugees than with immigrants and, as expected, that these experiences were less strongly related to perceived norms. Furthermore, the results observed in the four studies showed strong consistency, regardless of potential differences in methodological approach. Future studies should nevertheless further examine the potential impact of these levels of analysis in the investigated processes.

The different scales used across studies to assess participants’ initial outgroup attitudes constitute another limitation of the present research. One might argue that the scales used in Studies 2 and 4 assessing participants’ attitudes toward immigration policies are not equivalent to attitudes toward the outgroup. Yet, although we assessed initial attitudes in two different ways across the studies, the pattern of results remained the same. Thus, the findings observed whilst using different measures suggest that the underlying construct driving the effects is equivalent in essence, regardless of the operationalization of the Outgroup Attitude Scale. In addition, Visintin et al. (2018) demonstrated that there is a strong relationship between a country’s immigration policy and nationals’ attitudes toward immigrants. Specifically, countries with more exclusive immigration policies have higher levels of prejudice toward immigrants. Finally, scales measuring intergroup attitudes often include items on individuals’ agreement with policies aimed at improving (or not) the living conditions of the outgroup (e.g., Akrami et al., 2000). For these reasons, we are confident that the different outgroup attitude scales employed in the present studies reflect different ways of assessing intergroup attitudes, thus providing a convergent and valid approach to measuring participants’ initial attitudes toward refugees and immigrants. Nevertheless, future research should replicate our findings using a more traditional measure of outgroup attitudes.

The present findings may also be understood as supporting the hypothesis that ingroup norms moderate the effects of intergroup contact on intergroup relations. Indeed, there is an ongoing debate about whether intergroup contact is most effective in improving intergroup relations in inclusive or exclusive social contexts. Some research suggests that the positive effects of intergroup contact on intergroup relations appear specifically when exclusive (vs. inclusive) norms prevail, namely by limiting the influence of these norms (i.e., buffering effect of contact; e.g., Visintin et al., 2020). Conversely, other research suggests that intergroup contact is particularly effective in contexts where social norms are inclusive rather than exclusive (i.e., the galvanization effect; Borinca et al., 2021; Green et al., 2020; J. Kende et al., 2018). The present research provides evidence consistent with the galvanizing effect of inclusive norms, at least among prejudiced nationals, and relates this debate to research on individual differences suggesting that a galvanizing effect appears more easily among prejudiced individuals (Hodson et al., 2013). Yet, more research is needed to better understand when positive intergroup contact can result in a buffering or a galvanizing effect, for instance, as a function of contextual factors (e.g., J. Kende et al., 2021) and specific populations. Furthermore, as the current research focused on contact intentions of the national majority, future research should examine the extent to which these patterns occur among ethnic minorities (e.g., Wright & Baray, 2012).

## Conclusions

Our results suggest that policies conveying tolerant and inclusive injunctive norms, such as antidiscrimination laws, are not always sufficient to increase prejudiced nationals’ intentions to engage with refugees, especially in contexts where opportunities for intergroup contact are low. In such contexts, it is important to develop tools fostering indirect forms of contact to increase familiarity and prepare encounters with this outgroup.

## Notes

1. In the present paper, the term refugees also refers to asylum seekers. Moreover, while we do not deny the diversity of the refugee and immigrant groups on a multitude of dimensions and the generalization we make in addressing these two groups as general categories, our distinction is based on legal definitions. According to the 1951 Refugee Convention (UNHCR, 2011), a refugee is a person who, “is outside his or her country of nationality or habitual residence; has a well-founded fear of being persecuted because of his or her race, religion, nationality, membership of a particular social group or political opinion; and is unable or unwilling to avail him- or herself of the protection of that country, or to return there, for fear of persecution.” A migrant is a person “moving from one country to another for reasons other than persecution. Migrants may be fleeing dire poverty, or may be well-off and merely seeking better opportunities, or may be migrating to join relatives who have gone before them” (Sengupta, 2015).

2. We also asked participants to estimate the proportion of French nationals having contact with each target group (from 0 to 100%). Participants perceived a lower percentage of the French population as having contact with refugees (*M* = 25.44, *SD* = 17.60) compared to immigrants (*M* = 48.16, *SD* = 26.70), *F*(1, 118) = 96.78, *p* < .001, η_p_^2^ = .45. As the results for this measure were similar to those observed for the other two measures of normative perception, for brevity, we did not describe these results in the text.

3. We are grateful to Olivier Renaud for his help in conducting this statistical comparison. The R code for this test can be obtained from the first author upon request.

4. To estimate which norm condition significantly differed from the other conditions, we conducted a 2 (contact: positive = +1, control = −1) × 3 (ingroup norm: inclusive = +1, control = 0, exclusive = −1) x Initial Attitude Toward Refugees (standardized values) ANCOVA with positive intergroup orientations as the dependent variable. This analysis yielded the predicted Imagined Contact × Ingroup Norm × Initial Attitude interaction, *F*(2, 231) = 3.49, *p* = .032, η_p_^2^ = .03. Among unprejudiced nationals, the Imagined Contact × Ingroup Norm interaction was not significant, *F*(2, 231) = 0.62, *p* = .540, but it was among prejudiced nationals, *F*(2, 231) = 5.60, *p* = .004, η_p_^2^ = .05. Simple effects showed that, in the positive imagined contact condition, positive intergroup orientations toward refugees were higher in the inclusive norm condition compared to the exclusive and control conditions, *F*(2, 231) = 7.19, *p* = .008, η_p_^2^ = .03 and *F*(2, 231) = 9.86, *p* = .002, η_p_^2^ = .04, respectively. The exclusive condition did not differ from the control condition, *F*(2, 231) = 0.68, *p* = .401. Moreover, in the control (without imagined contact) condition, the exclusive ingroup norm increased positive intergroup orientations toward refugees compared to the control norm condition, *F*(2, 231) = 4.46, *p* = .036, η_p_^2^ = .02. The inclusive norm condition did not differ from the exclusive or the control norm conditions, *F*(2, 231) = 3.83, *p* = .052 and *F*(2, 231) = 0.17, *p* = .680, respectively.
